# Testing for *Within × Within* and *Between × Within* Moderation using Random Intercept Cross-Lagged Panel Models

**DOI:** 10.31234/osf.io/wktrb

**Published:** 2022-08-05

**Authors:** Lydia Gabriela Speyer, Anastasia Ushakova, Sarah-Jayne Blakemore, Aja Louise Murray, Rogier Kievit

**Affiliations:** a Department of Psychology, University of Cambridge, Cambridge, United Kingdom; b Department of Psychology, University of Edinburgh, Edinburgh, United Kingdom; c Center for Health Informatics, Computing and Statistics, University of Lancaster Medical School, United Kingdom; d MRC Cognition and Brain Sciences Unit, University of Cambridge, Cambridge, United Kingdom; e Cognitive Neuroscience Department, Donders Institute for Brain, Cognition and Behavior, Radboud University Medical Center, Nijmegen, The Netherlands

**Keywords:** RI-CLPM, moderation, Millennium Cohort Study, emotional problems, conduct problems

## Abstract

Random-Intercept Cross-Lagged Panel Models allow for the decomposition of measurements into between- and within-person components and have hence become popular for testing developmental hypotheses. Here, we describe how developmental researchers can implement, test and interpret interaction effects in such models using an empirical example from developmental psychopathology research. We illustrate the analysis of *Within × Within* and *Between × Within* interactions utilising data from the United Kingdom-based Millennium Cohort Study within a Bayesian Structural Equation Modelling framework. We provide annotated Mplus code, allowing users to isolate, estimate and interpret the complexities of within-person and between person dynamics as they unfold over time.

In behavioural and psychological studies, researchers are often interested in whether longitudinal relations between two variables are influenced by moderating factors. For example, one may be interested in whether positive maternal parenting behaviours are associated with a stronger (or weaker) relationship between maternal and child mental health (Suveg et al., 2011) or whether cognitive abilities influence the association between work stress and negative affect (Hyun et al., 2017). Among longitudinal data analyses methods, the cross-lagged panel model (CLPM) has been the method of choice when more than one wave of panel data is available (Biesanz, 2012). However, CLPMs suffer from a major limitation, that is, they conflate within- and between-person effects (Hamaker et al., 2015), making it difficult to isolate the within-person effects that tend to be of primary interest to developmental theory and interventions. Hamaker et al., for example, demonstrated that, if the variables in the CLPM exhibit stable individual differences (which is the case for the vast majority of psychological variables), then the cross-lagged estimates can be biased as estimates of within-person effects. More recently, an extension to the CLPM that overcomes this limitation has been proposed – the Random Intercept CLPM (RI-CLPM; Hamaker et al., 2015). If at least three waves of data are available, the RI-CLPM allows for the decomposition of observed data into within- and between-person components, thus ensuring that the cross-lagged effects, under certain assumptions, provide unbiased estimates of within-person effects (Hamaker et al., 2015). Similar to CLPMs, RI-CLPMs can be extended to include mediation (Speyer et al., 2021) or between-person moderation involving categorical variables in multi-group models (Mulder & Hamaker, 2020). However, moderation involving continuous variables, often described as interactions, are less straightforward to implement, for reasons discussed below.

In the current paper, we provide researchers with a guide to incorporating continuous interaction effects into RI-CLPMs using an empirical example from developmental psychopathology research. First, we built baseline models investigating the longitudinal within-person associations between conduct problems and emotional problems over three waves of data collected in children aged 3, 5 and 7 years. Second, we investigated whether between-person differences in cognitive abilities moderated within-person developmental cascades from conduct problems to emotional problems, thereby illustrating *time-invariant Between × Within* interactions. Third, we investigated a *time-varying Between × Within* interaction. Specifically, we tested whether between-person differences in peer problems (which vary between people as well as within people over time), moderated within-person developmental cascades. Finally, we analysed whether within-person deviations from a child’s general level of peer problems moderated cascades from conduct problems to emotional problems, thus illustrating *Within × Within* interactions.

Analyses were implemented in the structural equation modelling (SEM) software Mplus (L. K. Muthén & Muthén, 2018). This is the only software that can currently effectively deal with multiple latent variable interactions (Ozkok et al., 2021). To date, only one previous study has implemented continuous interaction terms within a RI-CLPM framework. Ozkok et al. (2021) illustrated latent variable interactions within the context of the relations between work conflict and job satisfaction, comparing results from *between × within*, and *within × within* interactions in a standard CLPM to results from a model controlling for stable between-person effects. Here we move beyond this by making the important distinction between *between* × *within interactions using a time-invariant moderator* vs *between × within interactions using the between-person component of a time-varying moderator*, as well as developing a *within × within* interaction involving two time-varying dependent variables. To achieve these goals, we must first tease apart the longitudinal within versus between person processes.

## The Random-Intercept Cross-Lagged Panel Model

Proposed by Hamaker et al. (2015), the RI-CLPM builds on the traditional CLPM by decomposing for a minimal example, two repeatedly measured observed variables (*x* and *y*) for persons *i* into three components (Equations 1 and 2 for *x* and *y* respectively):

(1) $$x_{it}=\mu_{xt}+x_{Bi}+x_{Wit}$$

(2) $$y_{it}=\mu_{yt}+y_{Bi}+y_{Wit}$$

**Figure 1:**
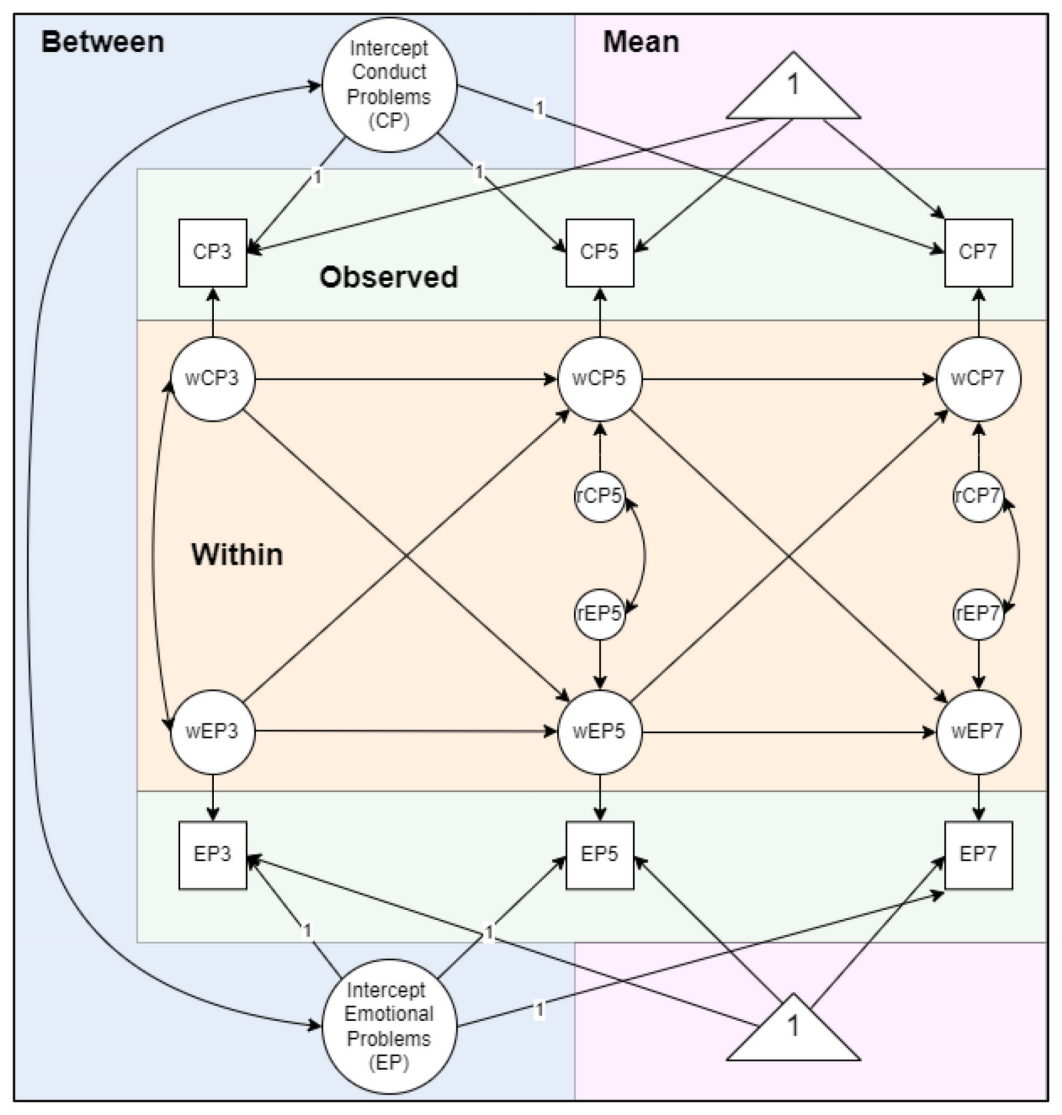
Illustration of a 3-Wave Bivariate RI-CLPM. Circles represent latent variables, squares observed variables and triangles represent means. r = residual, w = within-person.

Subscripts *i* and *t* represent vectors of values for individuals and time, respectively. First, we have the time-specific means (*μ_xt_, μ_yt_*), which reflect the temporal group means. These time-specific means are estimated as the means of all participants per timepoint and may be time-varying or time-invariant. Second, we have the between-person components (i.e. random intercepts, *x_Bi_*, *y_Bi_*), which capture the (stable) within person deviations from these group means. Random intercepts capture the stable between-person components (*x_Bi_*, *y_Bi_*) and represent an individual’s time-invariant deviations from the time-specific mean, using similar notation as in Ozkok et al (2021). In SEM software such as Mplus, the person-specific intercepts are specified using latent variables composed of repeated measures with factor loadings fixed to 1. Finally, we have the within-person components (*x_wit_*, *y_wit_*), which reflect the person specific deviations from their expected means at any one occasion. These terms (*x_wit_*, *y_wit_*), will be modelled using an autoregressive/cross-lagged structure later on. The within-person components (*x_wit_*, *y_wit_*), reflect the deviations of an individual’s observed scores from their expected score (comprised of time-specific means and person-specific random intercepts), thus capturing within-person fluctuations in a repeatedly measured variable. The within-person components are created by specifying a latent variable based on the observed variable and constraining the observed variables measurement error variances to 0 (Mulder & Hamaker, 2020). Constraining the residual measurement variances to 0 is generally recommended in order to clearly decompose observed scores into within- and between components (Ozkok et al., 2021). However, the requirement of zero residual variances can be relaxed without introducing substantial parameter bias to improve estimation in models with convergence difficulties provided that its value is fixed to be reasonably small (Asparouhov & Muthén, 2020; Ozkok et al., 2021). In bivariate or RI-CLPMs, random intercepts (*x_Bi_*, *y_Bi_*) of different repeatedly measured variables are typically allowed to co-vary in order to capture the associations between their general (i.e. time-stable) levels, see Figure 1. This is critical for avoiding the bias seen in the CLPM because it allows the individual differences to covary and thus not contaminate the within-person relations.

Once the observed scores have been decomposed into within- and between-person components, autoregressive (*β^x^_Wx_, β^y^_Wy_*) and cross-lagged (*β^x^_Wy_, β^y^_Wx_*) effects can be specified between the within-person components, with *u^x^_Wit_* and *u^y^_Wit_* further representing time-specific random changes in *x* and *y* (Equations 3 and 4).

(3) $$x_{Wit}=\beta^{x}{\;}_{wx}\times x_{it-1}+\beta^{x}{\;}_{wy}\times y_{W_{it-1}}+u^{x}{\;}_{W_{it}}$$

(4) $$y_{Wit}=\beta^{y}{\;}_{wy}\times y_{W_{it-1}}+\beta^{y}{\;}_{wx}\times x_{W_{it-1}}+u^{y}{\;}_{W_{it}}$$

The autoregressive path captures the extent to which a person’s time-specific deviation of their own expected score persists across additional waves (inertia). The cross-lagged structure captures, for example, that an individual experiencing more peer problems than their own average is likely to have higher, or lower, emotional problems at a later timepoint. Finally (residual) covariances are included to reflect contemporaneous within-person (residual) associations (see Figure 1). For further discussion of RI-CLPMs, see Hamaker et al. (2015).

## The Moderated Random-Intercept Cross-Lagged Panel Model

### Between × Within interaction using a time-invariant moderator

These cross-lagged effects can capture dynamic processes, that is changes over time. However, a researcher may have reason to believe that these effects vary over time (e.g. Orben et al., 2022), or differ according to the levels of some other factor that varies between people, such as gender, cognitive ability, age or any other variable of interest. Such *moderation effects* can be modelled, and can operate either at the within-person or at the between-person level. We will discuss both in turn. Testing moderation using multi-group models where the moderator can be defined in terms of small number of groups has been discussed elsewhere (Mulder & Hamaker, 2020). However, most variables cannot be justifiably separated into distinct categories and doing so nonetheless (e.g. creating subgroups based on median splits) results in a loss of valuable information (MacCallum et al., 2002). A solution is to add *Between × Within* interactions of continuous variables into the RI-CLPM. This can be done by introducing moderating effects on the cross-lagged effects (Ozkok et al., 2021). Here, we introduce a moderator (***z_i_***) operating at the between-person level (Equation 5). We can represent *z_it_* as a time-specific mean *μ_zt_* and the between-component (*z_i_*).

(5) $$z_{it}=\mu_{zt}+z_{i}$$

In a time-invariant scenario, the within-person cross-lagged effect of, for example, conduct problems (***x_w_it-1__***) on emotional problems (***y_wit_***), can be thought of as the outcome variable that is predicted by a variable (**z**) that differs between people. If a variable is only measured once, it is not possible to decompose the variable into within and between components, leaving us only with ***z_it_*** = ***z_i_.*** In this case, interactions are composed of the within-person centred predictor and the observed time-invariant moderator (Equation 6).

(6) $$y_{Wit}=\beta^{y}{\;}_{wy}\times y_{W_{it-1}}+\beta^{y}{\;}_{wx}\times x_{W_{it}-1}+u^{y}{\;}_{W_{it}}+z_{i}(\beta_{B_{z}W_{x}}\times x_{W_{it}-1})$$

In Mplus, such interactions can be estimated by defining an interaction using the XWITH command and using this interaction term as a predictor of the within-person centred outcome variable (Asparouhov & Muthén, 2020). Of note, once latent interaction variables are specified within Mplus, standard model fit indices are no longer available. However, the inclusion of interaction terms does not usually alter model fit substantially, hence, it is recommended that researchers first to check model fit statistics in a baseline model before introducing interaction terms (Asparouhov & Muthén, 2020). If fit is acceptable or good in the model prior to including moderators, it will be unlikely that the full model fits poorly beyond the increased complexity of the added parameters.

### Between × Within interaction using the between-person component of a time-varying moderator

If a variable is not assumed to be stable across time and is measured at multiple time points, the variable can be decomposed into within- and between-person components by including a random intercept for the moderator variable (Ozkok et al., 2021). If this decomposition is not performed and the observed variable is directly included when defining interaction terms, moderation effects would represent a mixture of within- and between-person effects. For example, if interested in whether the link between external events and mood is stronger if a person consumes more alcohol than they do on average, it is necessary to account for differences in average levels of alcohol consumption. This is because consuming ‘less alcohol than average’ will be different for a person who on average tends to consume little alcohol compared to someone who generally consumes a lot. Conflating these within- and between-person effects should usually be avoided as this would bias parameter estimates of distinct mechanisms in a hard to predict direction. To address this challenge, the latent between-person variable, i.e. the random intercept, can be added as a moderator analogous to a time-invariant between-person factor by defining an interaction that is then used as a predictor of the within-person centred outcome variable (Ozkok et al., 2021). Of note, even though the observed moderator is treated as time-varying, the disaggregated between-person component of the moderator is treated as time-invariant and indicative of stable between-person differences. Hence, if interactions are specified across multiple lags, interaction terms are specified using the time-varying within-person centred predictor and the stable between-person component of a time-varying moderator separately for each lag (though equality constraints can be introduced in case the investigated effects are hypothesised to be stable across the observed timespan). Formally, the introduction of a time-varying moderator results in the same equation as for time-invariant moderators (6), however, *z_it_* should now decomposed into within (*z_W_it__*) and between components (*z_B_it__*) and Equation 6 can be extended to include an extra term for the within-person structure of the moderator.

(7) $$y_{Wit}=\beta^{y}{\;}_{Wy}\times y_{W_{it-1}}+\beta^{y}{\;}_{Wx}\times x_{W_{it}-1}+\beta^{y}{\;}_{Wz}\times z_{W_{it}-1}+u_{Wit}^{y}+z_{Bi}(\beta^{y}{\;}_{B_{z}W_{x}}\times x_{W_{it}-1})$$

### Within × Within interaction using the within-person component o f a time-varying moderator

If one is interested in whether within-person cross-lagged effects differ based on another within-person factor, a moderation effect needs to be introduced purely at the within-person level. For example, this would be appropriate for examining research questions such as whether higher levels of peer problems at one timepoint, relative to a child’s general levels of peer problems, exacerbates the association between conduct problems and later development of emotional problems. When examining time-intervals that span several years, these interaction effects should usually be treated as time-varying, as effects may differ based on the developmental period under investigation. However, interactions as well as cross-lagged and autoregressive effects can be equality constrained across time reflecting that investigated effects are assumed stable across the observed timespan (which may be done to reduce computational complexity). For *Within × Within* interactions, the moderator also needs to be decomposed into between- and within-components with the within-person component of the time-varying moderator being used in the definition of interaction terms (***β^y^_Wxz_***):

(8) $$y_{Wit}=\beta^{y}{\;}_{Wy}\times y_{Wit-1}+\beta^{y}{\;}_{Wx}\times x_{Wit-1}+\beta^{y}{\;}_{Wxz}\times x_{Wit-1}\times z_{Wit-1}+u^{y}{\;}_{Wit}$$

Using the XWITH command in Mplus, the interaction is defined between the moderator at time *t* and the predictor at time *t* and can then be used as a predictor of the outcome at time *t+1*. In all cases, the predictor *and* outcome are centred at the within-person level.

While we have discussed the estimation of *Within × Within* interactions separately from *Between × Within* interactions, these can be combined, allowing interpretations to focus on one or the other effect depending on the research question at hand. Formally, this entails combining Equations 7 and 8 to simultaneously estimate interactions involving the within-and the between-person component of the moderator:

(9) $$\begin{array}{c} y_{Wit}=\beta^{y}{\;}_{Wy}\times y_{W_{it-1}}+\beta^{y}{\;}_{Wx}\times x_{W_{it}-1}+\beta^{y}{\;}_{Wz}\times z_{W_{it}-1}+u^{y}{\;}_{Wit}+z_{Bi}(\beta_{B_{z}W_{x}}\times x_{W_{it}-1})\\ +\beta^{y}{\;}_{WxZ}\times x_{Wit-1}\times z_{Wit-1} \end{array}$$

In theory, estimating these effects simultaneously has a clear advantage as it allows for the examination of research questions about the effects of both the within- and between-person components of a time-varying moderator within one model, thus allowing for clearer comparisons between the two effects. In addition, including both the within- and between-components simultaneously would align more closely with the general approach taken by Mplus of separating within- and between-person components when estimating multilevel models. However, at this time, the computational resources required for estimating such a model make the implementation of *Within × Within* and *Between × Within* interactions within one model practically unfeasible, as discussed in more detail in the next section.

## Bayesian Estimation

One important computational consideration that needs to be considered when estimating latent variable interactions is that such analyses are highly computationally demanding, making it necessary to use numerical integration for model estimation (Asparouhov & Muthén, 2020; Preacher et al., 2016). While this is not usually a problem when only one interaction term is included, the number of integration points required increases with every subsequent interaction that is specified. Considering the longitudinal modelling context, most research questions likely require the specification of interaction terms not only on the cross-lagged effect from time 1 to time 2 or from time 2 to time 3, but across all lags involving the same variables. While these can be constrained to equality to reduce complexity, depending on the number of measurement occasions, the number of interactions and consequently integration points can still rapidly exceed the memory capacity of standard computing resources, making such analyses computationally unfeasible. A solution to this problem is the use of a Bayesian estimator (Asparouhov & Muthén, 2020; Ozkok et al., 2021), which, albeit still requiring a lot of computational resources, allows for the investigations of multiple latent variable interactions within one model.

Bayesian estimation combines the likelihood of the data with prior distributions for unknown parameters to obtain posterior distributions. Parameter estimates are then obtained by taking the mean or median of each parameter posterior distribution (van Ravenzwaaij et al., 2018). Credible intervals can then be used as an indicator of significance for regression slopes, interpreted alongside the effect size; they indicate the 95% probability that the true parameter estimate would lie within the interval, based on the evidence provided by the observed data. Given the Bayesian modelling context, priors can be set in order to allow for the incorporation of prior knowledge, but can also be specified to be diffuse, in which case the data dominates the results. To estimate the posterior distributions, parameters are sampled from conditional distributions following a Markov Chain Monte Carlo (MCMC) procedure (van Ravenzwaaij et al., 2018). Convergence can be checked using the criterion of potential scale reduction (PSR) values close to 1. This indicates whether the between-chain variation (relative to the total of between- and within-chain variation) of two (or more) Markov chains is close to zero (Asparouhov & Muthén, 2010; B. Muthén, 2010). In Mplus, PSR convergence criteria are calculated using 1 + ∈ where ∈ = *fc* with *f* representing a multiplicity factor that adjust the convergence criterion depending on the number of parameters in the model and *c* representing the convergence criterion that can be adjusted using the BCONVERGENCE argument. Per default, *c* = 0.0 5 which results in a PSR convergence criterion between 1.05 and 1.1 for most models estimated in Mplus (Asparouhov & Muthén, 2010). Once convergence is first achieved, the number of iterations should be doubled to ensure stable convergence of PSR values <1.1, which further allows relative bias to be examined. Relative bias here refers to a comparison of parameter estimates between a model based on first convergence and a model based on doubling the number of iterations, with <10% difference indicating little relative bias (McNeish, 2016).

Because using PSR as a convergence criterion may not result in optimal precision of parameter estimates, recent work has suggested alternative strategies for robust Bayesian estimation using a greater number of iterations and a stricter threshold based on monitoring Effective Sample Size (ESS) (e.g., Zitzmann et al., 2021; Zitzmann & Hecht, 2019). However depending on the model and dataset, this approach may lead to prohibitively long estimation time (months) and excessive computational burden (cf. Lannelongue et al., 2021), so the best trade off between further gains in precision of parameter estimates and standard errors versus computational demands will vary with context. We therefore proceeded here using classical thresholds (as advocated in Asparouhov & Muthén, 2010; Depaoli & van de Schoot, 2017; Gelman et al., 2004), which converged in a reasonable time (days), and showed virtually identical results after doubling of the number of iterations.

If no informative priors are specified, as in the examples in the current study, Mplus makes use of diffuse priors to allow the data to dominate the posterior, in which case, the results should closely resemble results of standard maximum likelihood estimation (Asparouhov & Muthén, 2010). In baseline models without interactions, Bayesian equivalents of standard model fit indices such as Comparative Fit Index (CFI), Tucker Lewis Index (TLI) and Root Mean Square Error of Approximation (RMSEA) are also available and can be interpreted analogous to the model fit indices available when using standard maximum likelihood estimation (Asparouhov & Muthén, 2021). For a thorough introduction to Bayesian Analysis in Mplus, see (Asparouhov & Muthén, 2010, 2020; B. Muthén, 2010). Simulations of latent variable interactions in CLPMs (including *between × between*) are available in Ozkok et al (2021). Next, we illustrate how this modelling framework can be applied in practice.

## Empirical Example

An estimated 10-20% of children and adolescents experience mental health problems (WHO, 2018) and nearly half of these children will have, or go on to develop, at least one other mental health problem (Caspi et al., 2020). Various theories have attempted to explain the high co-occurrence of mental health difficulties, including network models of psychopathology, focusing on the direct relations between different mental health symptoms (Borsboom & Cramer, 2013), and developmental cascade models focusing on the longer term associations between different types of mental health issues (Masten & Cicchetti, 2010). Developmental cascade models propose that co-occurring mental health problems are the result of within-person directional relations from one mental health problem to another. For instance, conduct problems may increase the likelihood of other mental health problems over development via problems with peers or lower academic achievement, which might in turn lead to a lack of self-esteem and later emotional problems (Capaldi, 1992). Studies have found evidence for developmental cascades from externalising problems to internalising problems (e.g., Murray et al., 2020). However, few studies have tested such cascades within a framework that allows for an appropriate disaggregation of within-person effects (for an example, see Speyer et al., 2021), and no studies have investigated whether the strength of such cascade effects may be affected by continuous moderating factors. In line with a risk-protective model (Rutter, 1979), factors such as cognitive abilities or peer problems may buffer or increase an individual’s risk for the development of co-occurring mental health problems. For example, studies have suggested that aggressive children with peer problems are more likely to develop additional internalising problems than children with healthy peer relationships (Fite et al., 2014). However, such studies have primarily focused on comparing rank order changes between people primarily using cross-sectional designs, even though such processes are hypothesised to unfold over time at the within-person level.

To date, testing the effect of moderating factors on within-person developmental cascades has been challenging as classic methods such as moderated cross-lagged panel analyses do not allow for an appropriate disaggregation of within- and between-person effects. However, investigating such questions is an important avenue for future research as it would enable clearer insights the mechanisms underlying cascades leading to co-occurring mental health problems, and a closer mapping of theories and hypotheses onto our formal tests. Here, we illustrate how one can test whether peer problems and cognitive abilities moderate developmental cascades from conduct to emotional problems using three waves of data (N = 13955; ages 3, 5 and 7) from the Millennium Cohort Study (MCS).

### Participants

Participants were the 13955 children who participated up to the age 7 sweep of the MCS. The MCS is a UK based longitudinal birth cohort that has been following the lives of around 19000 children and their families from shortly after birth at the start of the 21^st^ century across seven data collection waves (Connelly & Platt, 2014). The MCS was approved by the London Multicentre Research Ethics Committee. Participating parents gave written consent at each sweep. Detailed information on the MCS is available elsewhere (Connelly & Platt, 2014).

### Measures

Conduct problems, emotional problems and peer problems were measured using the Strength and Difficulties Questionnaire (SDQ; Goodman, 1997) at ages 3, 5 and 7. Five items each, scored on a 3-point scale are summed up to derive subscale scores with higher scores indicating more problem behaviours (range 0-10). Psychometric evaluations of the SDQ have generally found favourable results (Kersten et al., 2016), including longitudinal invariance across ages 5 to 14 in the MCS sample used here (Murray, Speyer, et al., 2021). Cognitive abilities were measured at age 3 using the composite percentile score on the Bracken School Readiness Assessment, assessing understanding of colours, letters, numbers, sizes, comparisons, and shapes (Bracken, 2002). For descriptive statistics including trajectory plots see the online supplementary Tables S1 and Figures S1-S3.

## Statistical Analysis

### Baseline RI-CLPM

Following the procedure outlined above, we first a baseline RI-CLPM was fit to investigate whether conduct problems and emotional problems are reciprocally related at the within-person level. Random intercepts were estimated for conduct problems and emotional problems and cross-lagged and autoregressive effects were specified on the within-person centred residuals, see Figure 1. To check the model fit of a baseline model also including the within-person moderator peer problems, a second baseline RI-CLPM was fit, which included the decomposition of peer problems into within- and between-components and included the within-person centred peer problems component in the cross-lagged and autoregressive structure. The baseline models could have been analysed using standard maximum likelihood estimation, but, for better comparability to the moderation models, they were also estimated using a Bayesian estimator. Model fit was judged to be good if CFI and TLI were > .95 and RMSEA was < .06 (Hu & Bentler, 1999). Model convergence was based on PSR values remaining stable below 1.1. In order to check that PSR values did not randomly fluctuate close to 1 (indicating unstable estimates), we doubled the number of iterations once first convergence was achieved. This further allowed us to check for relative bias in parameter estimates. To aid convergence, measurement error variances were fixed to 0.2. The same value was chosen for all variables at each time-points as all repeatedly measured variables were on the same scale. Generally, this specification should not lead to substantial parameter bias as long as measurement error variances are a reasonable approximation of zero (Asparouhov & Muthén, 2020; Ozkok et al., 2021). When using Bayesian estimation in Mplus, missing data are treated as additional unknown quantities, thus, they are sampled from their conditional posterior distribution (McNeish & Hamaker, 2019). Annotated Mplus code and full model results for all conducted analyses are available on OSF: https://osf.io/tjxrd/?view_only=3049a194340340f892dfe0ed055e040a.

### Between × Within interaction using a time-invariant moderator

To analyse whether between-person differences in cognitive abilities moderated within-person developmental cascades from conduct problems to emotional problems, cognitive abilities were added to the baseline model. In particular, cognitive abilities at age 3 were used to define interaction terms between the within-person deviations in conduct problems at age 3 as well as at age 5. In turn, these interaction terms were used as predictors of within-person deviations in emotional problems at age 5 and age 7. As cognitive abilities were only measured once, no decomposition into within- and between-person components was performed. Importantly, this assumes that cognitive abilities are stable at the within-person level, even though cognitive abilities do tend to change across early- to middle-childhood. Thus, as this assumption does not hold, estimates will represent a mix of within- and between-person moderation effects. The model was estimated using a Bayesian estimator with PSR values below 1.1 used as the convergence criterion. For time-invariant moderators (that are only used as predictor variables in the model), missing data is treated using listwise deletion, thus sample size was reduced to 11516 cases. Mplus code is available on OSF.

### Between × Within interaction using the between-person component of a time-varying moderator

For the analysis of the effect of between-person differences in peer problems, that is, general levels of peer problems, on the within-person relations between conduct problems and emotional problems, peer problems were decomposed into a within-person and a between-person component as outlined for the baseline RI-CLPM. The between-person component, that is, the random intercept, was then used to define interaction terms. Analogous to a time-invariant moderator, the interaction term was subsequently included in the model as a predictor of within-person changes in emotional problems at age 5 and age 7.

### Within × Within interaction using the within-person component o f a time-varying moderator

Finally, to test whether within-person changes in peer problems moderated the within-person relations between conduct problems and emotional problems, the within-person centred components of peer problems were used to define interaction terms. Interactions between peer problems and conduct problems at age 3 and at age 5 and were used as a predictor of emotional problems at age 5 and age 7 respectively. As for the analysis of *Between × Within* interactions, model convergence was checked by confirming that PSR values were close to 1.

## Results

### Baseline RI-CLPM

**Figure 2:**
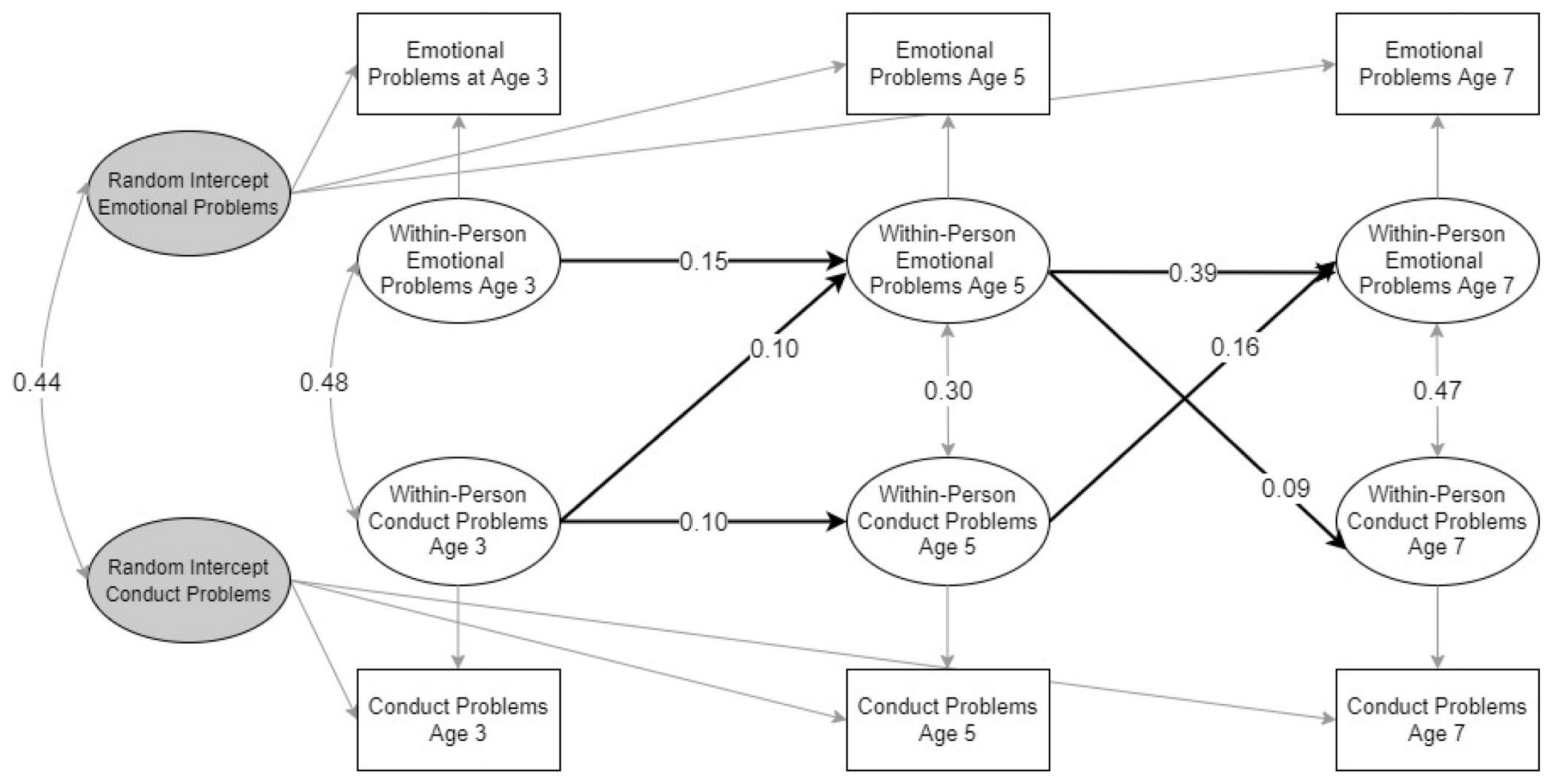
RI-CLPM for emotional and conduct problems. Only statistically significant autoregressive and cross-lagged paths are shown. Estimates are unstandardised.

The baseline model including only conduct problems and emotional problems had good fit with CFI = .995, TLI = .955, RMSEA = .065 and stable convergence of PSR values below 1.1 with relative bias for parameters of interest suggested to be low. The model indicated that conduct problems and emotional problems shared reciprocal within-person relations across ages 5 to 7 with conduct problems predicting an increase in emotional problems and vice versa. Across ages 3 to 5, conduct problems were associated with an increase in emotional problems. For a summary of significant cross-lagged and autoregressive effects, see Figure 2.

**Figure 3:**
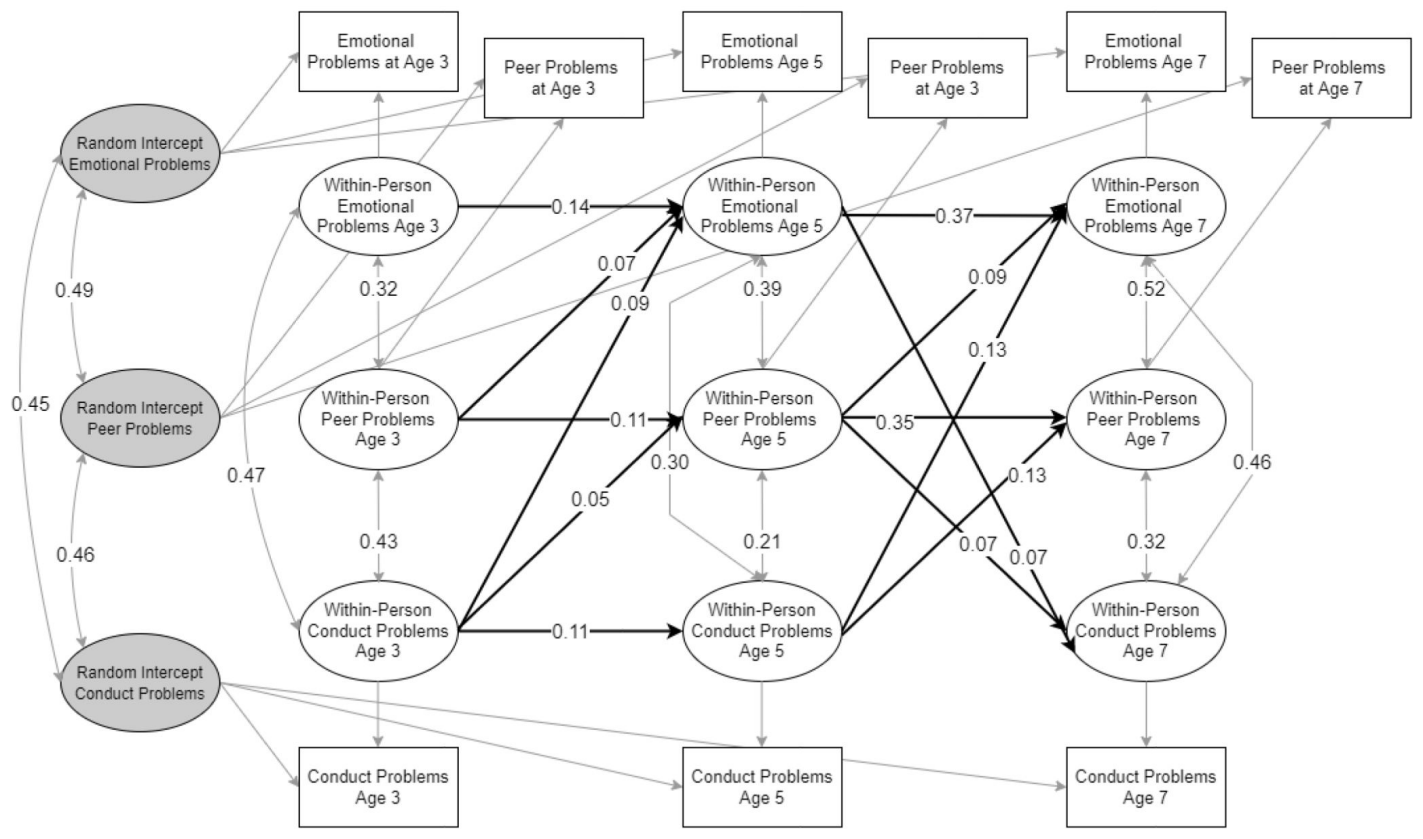
RI-CLPM for emotional and conduct problems. Only statistically significant paths are shown. Estimates are unstandardised.

The baseline model including peer problems also showed good fit (CFI = .996, TLI = .945, RMSE = .060) and stable convergence of PSR values. Results were in line with the baseline model and further suggested that, at age 3 and age 5, within-person deviations in peer problems were associated with increases in within-person levels of emotional problems at age 5 and 7, and at age 5, with increases in conduct problems at age 7. Conduct problems at ages 3 and 5 were further associated with within-person increases in peer problems at ages 5 and 7. See Figure 3 for a summary of significant effects.

### Between × Within interaction using a time-invariant moderator

**Figure 4:**
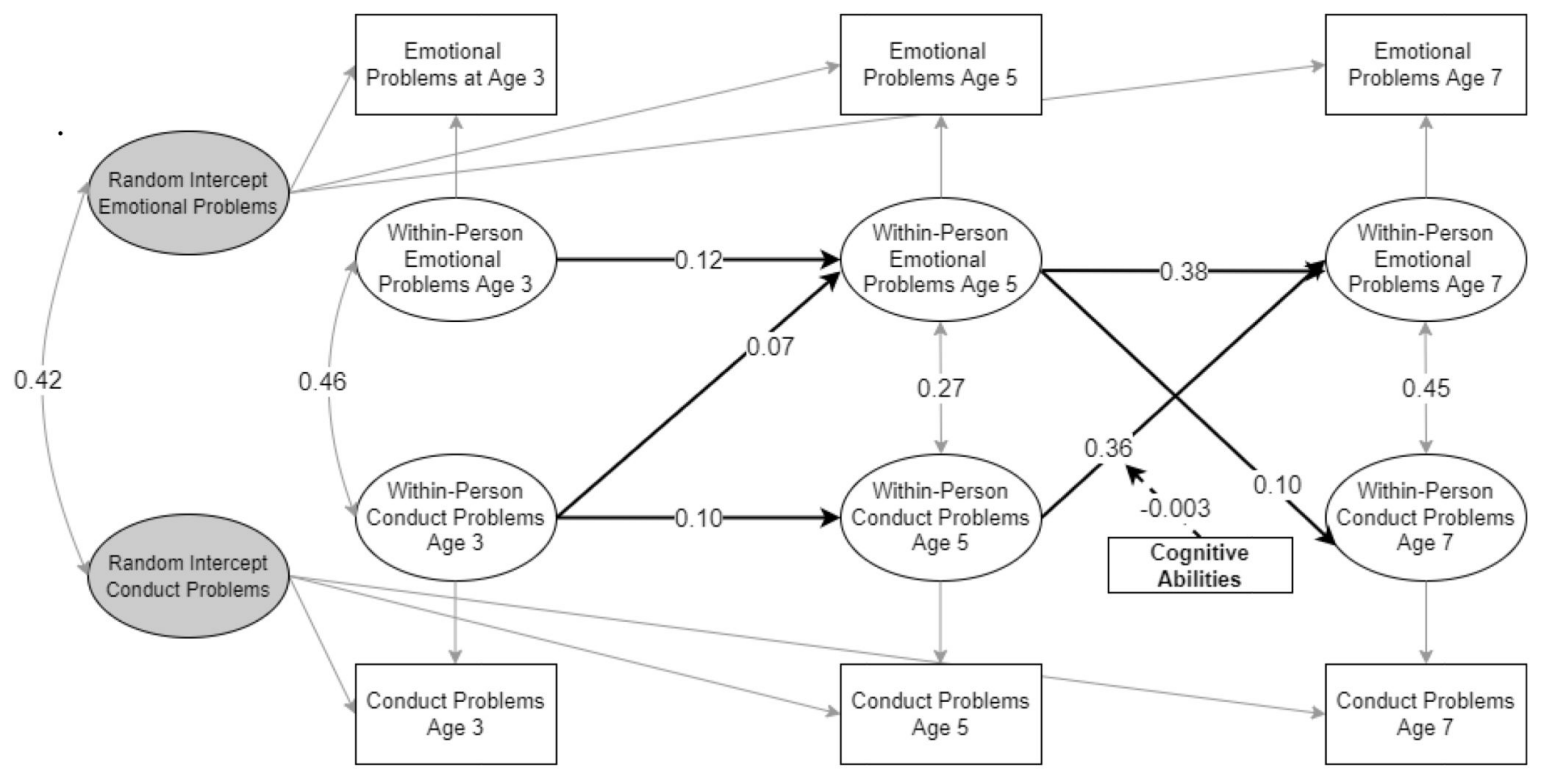
RI-CLPM using cognitive abilities as a time-invariant moderator. Only statistically significant paths are shown. Estimates are unstandardised.

Results of the model investigating whether cognitive abilities moderated within-person developmental cascades from conduct problems to emotional problems indicated that cognitive abilities had a significant moderating effect on the within-person cross-lagged effect of conduct problems at age 5 to emotional problems at age 7. In particular, the results suggested that higher cognitive abilities were associated with a decrease in the strength of the association between conduct problem and later within-person increases in emotional problems. This effect was not observed for the developmental cascade from age 3 to age 5. A summary of significant effects is visualised in Figure 4. Parameter estimates and corresponding credible intervals are visualised in Figure 7 and reported in Table S2 in the online supplementary, showing that the inclusion of interaction terms tends to slightly reduce the magnitude of effects observed in the baseline model.

### Between × Within interaction using the between-person component of a time-varying moderator

**Figure 5:**
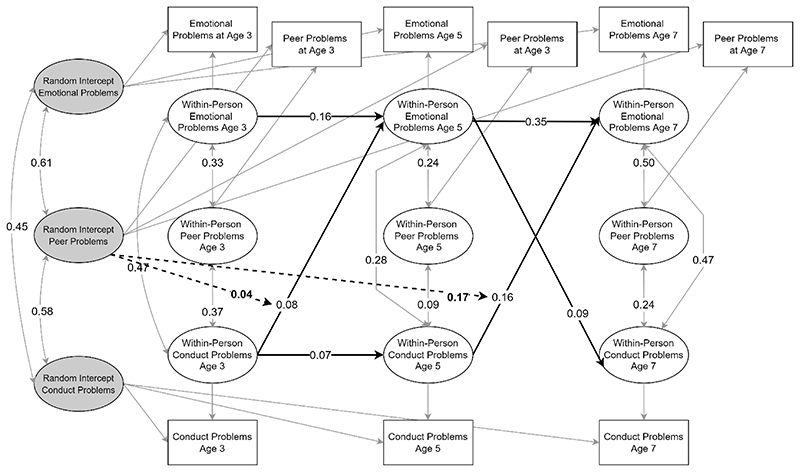
RI-CLPM using the between-person component of peer problems as a moderator. Only statistically significant paths are shown. Estimates are unstandardised.

Investigating the effect of stable between-person difference in peer problems, results suggested that higher general levels of peer-problems were associated with stronger within-person cascades from conduct problems to emotional problems across ages 3 to 5 and 5 to 7. For a summary of significant effects, see Figure 5. For parameter estimates and corresponding credible intervals, see Figure 8 and Table S2 in the online supplementary. Due to computational resource constraints, number of iterations were not doubled for this model.

### Within × Within interaction using the within-person component o f a time-varying moderator

**Figure 6:**
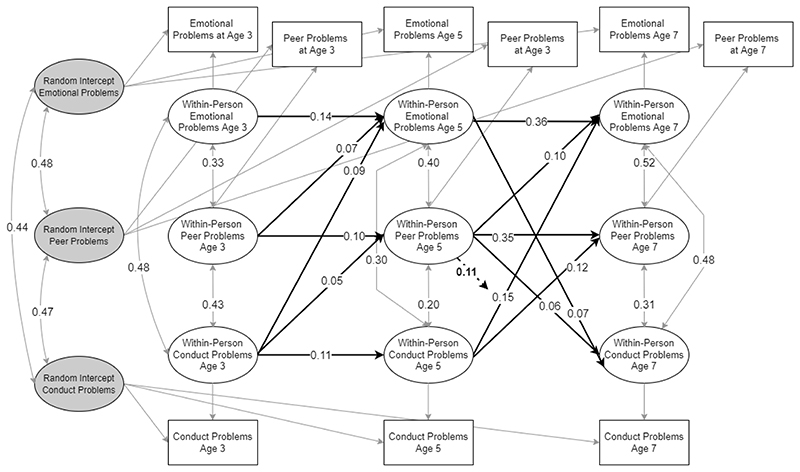
RI-CLPM using the within-person component of peer problems as a moderator. Only statistically significant paths are shown. Estimates are unstandardised.

**Figure 7:**
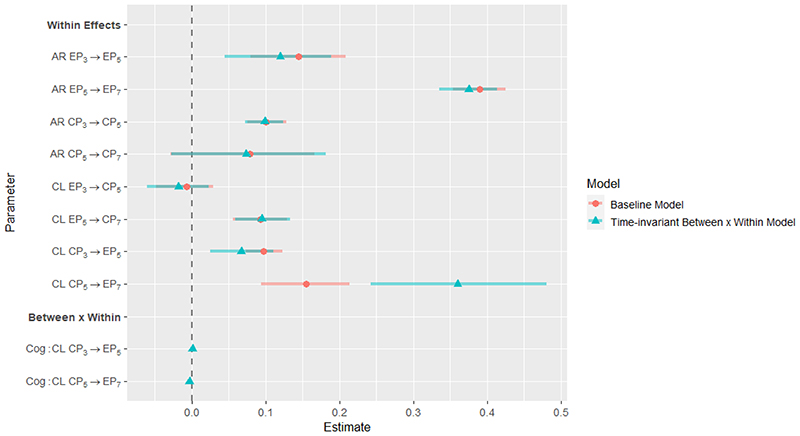
Parameter estimates for Baseline Model and Time-invariant *Between × Within* Model. Grey bars represent credible intervals.

**Figure 8:**
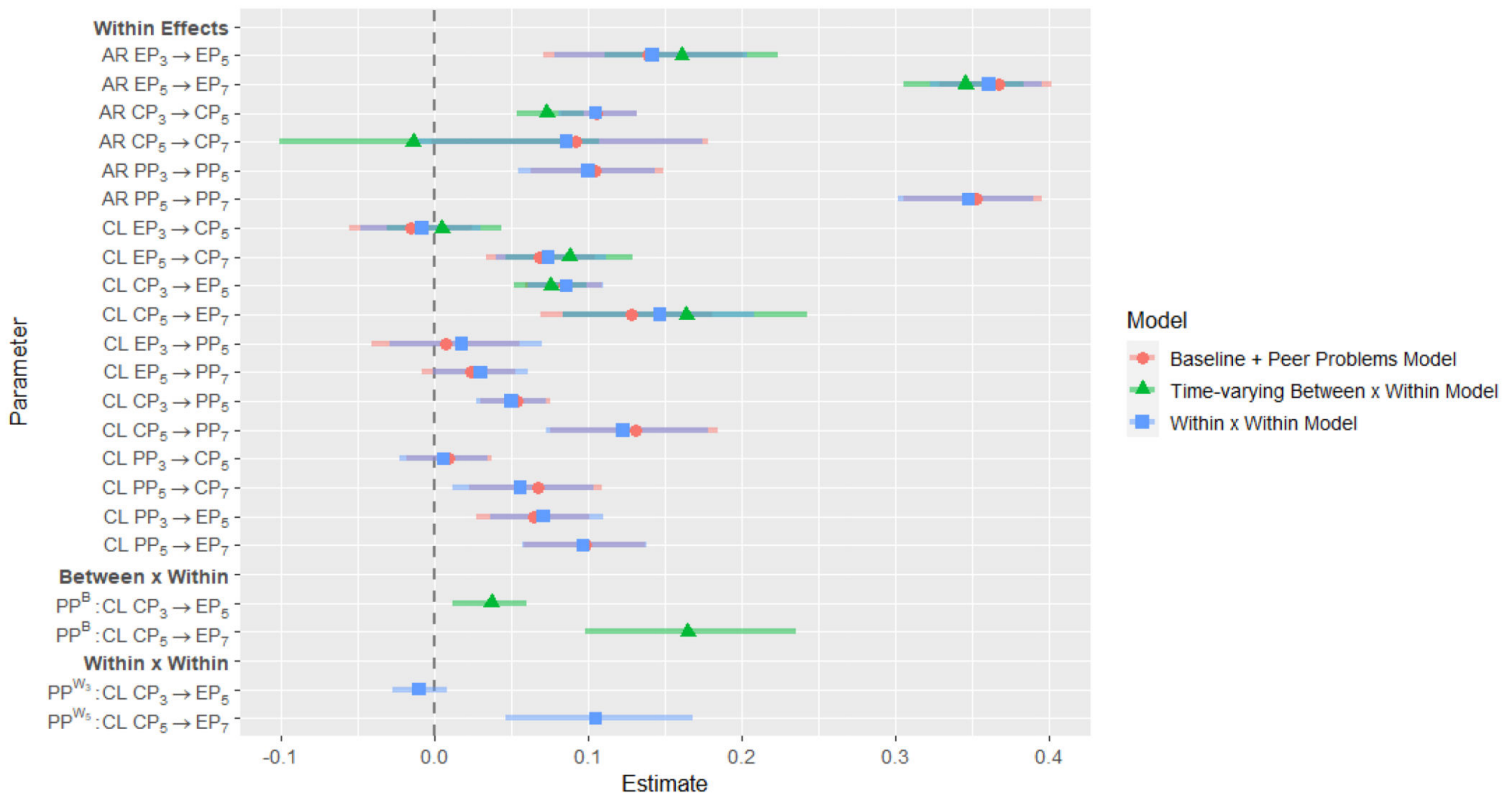
Parameter estimates for Baseline + Peer Problems Model, Time-varying *Between × Within* Model and *Within × Within* Model. Grey bars represent credible intervals.

Finally, investigating whether within-person deviations from general levels of peer problems moderated within-person developmental cascades from conduct problems to emotional problems, results suggested that the within-person component of peer problems significantly moderated a cascade from conduct problems at age 5 to emotional problems at age 7. Higher peer problems at age 5 relative to a child’s general levels of peer problems were associated with stronger within-person effects of conduct on emotional problems. A summary of significant effects is visualised in Figure 6. Parameter estimates and corresponding credible intervals are visualised in Figure 7 and reported in Table S2 in the online supplementary.

## Discussion

The aim of the present paper was to illustrate the analysis of *Between × Within* and *Within × Within* interactions within a RI-CLPM framework using Mplus. To illustrate the implementation procedures for estimation of different interaction effects that may be of interest to researcher, we used an empirical example from developmental cascades research. Specifically, we investigated whether within-person deviations from average levels of peer problems as well as between-person differences in peer problems and cognitive abilities moderated longitudinal within-person cascades from conduct problems to emotional problems. Empirical results suggested that cognitive abilities, as well as within-person deviations from general levels of peer-problems, as well as the general levels of peer problems, moderated within-person developmental cascades from conduct problems at age 5 to emotional problems at age 7, with general levels of peer problems further moderating a cascade from age 3 to age 5. Overall, the results suggest that the analysis of moderation effects has the potential to increase our understanding of the processes underlying cascades leading to the development of co-occurring mental health problems. Such analyses are likely to be of benefit to a range of researchers conducting analyses using longitudinal data. In particular, moderation analyses within a RI-CLPM framework may not only aid the testing of existing hypotheses but also encourage the consideration of moderation effects in developmental theory since the availability of new modelling methods can lead theoretical innovations just as much as theoretical innovations can drive methodological developments. Moreover, we find that the inclusion of such interaction parameters affects the estimates of other parameters in the model, thus, suggesting that they are not only of interest in their own right but may in fact improve the accuracy of the model in its entirety.

When interested in moderation effects within a longitudinal context, the prime question to consider is whether such interactions are present at the within-, the between- or at both the within- and between-person level. If one is interested in how factors that are stable over time but differ between people influence the within-person effects of one variable on another, *Between × Within* interactions using a time-invariant moderator can be used to gain an increased understanding of such dynamics. If a variable is not stable over time, for instance, as it fluctuates due to occasion-specific influences, but it is purely the between-person component i.e. stable aspect, that is of interest, then the observed variable has to be decomposed into a within- and between-component. This ensures that the interaction effect only represents the effect of general (i.e. stable) differences and not a mixture of within- and between-person effects (Ozkok et al., 2021). If one is interested in whether deviations from a person’s general levels of a variable moderate the within-person associations between two variables, then interactions need to be specified purely at the within-person level. Once the correct level of analysis has been identified based on theory, these interaction effects can be implemented and estimated in statistical packages. At present, the computational demands on estimating these parameters are such that a dedicated software (Mplus) is needed to achieve stable and robust estimation. We have included fully commented examples of the analyses presented here on OSF to allow others to apply these techniques in novel applications.

Results of our empirical example offer insights into factors influencing the dynamic effects of conduct on emotional problems. First, the results support prior research finding evidence for developmental cascades from conduct to emotional problems (Capaldi, 1992; Murray et al., 2020). Evidence for cascades in the other direction has been mixed (Carlson & Cantwell, 1980; Speyer et al., 2021), however, findings of the current study suggest that these cascades may be at play during middle childhood.

Examining the effect of moderators, we found that cognitive abilities had a small but protective effect on a within-person cascade from conduct to emotional problems, with children with higher cognitive abilities showing smaller increases in emotional problems at age 7 following prior increases in conduct problems at age 5. Prior research has suggested that higher cognitive abilities may protect children against internalising problems (Riglin et al., 2016), while lower cognitive abilities have been associated with increased co-occurring mental health problems (Fuhrmann et al., 2021; Koenen et al., 2009). To our knowledge, this is the first study finding that the directional within-person effects between two different mental health problems indeed differ based on a child’s cognitive abilities. Findings suggest that children with lower cognitive abilities suffering from conduct problems are at increased risk of developing emotional problems, thus, clinicians may need to pay close attention to such children to prevent the development of co-occurring problems.

For peer problems, we found that deviations from a child’s average levels of peer problems moderated a cascade from conduct to emotional problems. Specifically, findings suggested that children experiencing higher levels of peer problems compared to their normal levels are at increased risk of developing emotional problems at age 7 following increases in their conduct problems at age 5. Children exhibiting generally higher levels of peer problems are also at increased risk of developing emotional problems as a result of increases in conduct problems. This was evidenced by significant moderation effects of general levels of peer problems on cascades from conduct to emotional problems. Peer problems have commonly been investigated as a mediator in the relations between conduct problems and emotional problems (Capaldi, 1992; Murray, Obsuth, et al., 2021), but some studies have also suggested that they may act as a *moderator*, for instance, by exacerbating the relations between aggressive behaviours and depression (Fite et al., 2014). Results here offer evidence that within-person deviations in peer problems as well as general levels of peer problems are associated with increases of the within-person effects of conduct on emotional problems.

Overall, results of this empirical example highlight the potential of investigating *Between × Within* and *Within × Within* interaction effects within RI-CLPMs for testing new hypotheses in developmental psychology. However, a number of practical limitations have to be considered. First, while the use of a Bayesian estimator makes estimation of models including latent variable interactions computationally feasible, convergence time is still fairly extensive (ranging from hours to days), especially when going beyond more than two interactions and using large sample sizes. Future research is needed to reduce convergence times as computation for the interaction analyses presented here may still exceed the computational resources of standard office computers when analysing a larger number of waves including multiple interaction terms. One such avenue may be the respecification of SEMs as *computation graphs*, which allow for the more easy integration of optimization tools from machine learning (van Kesteren & Oberski, 2019). Multilevel factor score approaches may also be of use in this context as they have already been shown to work for latent variable interactions (Devlieger & Rosseel, 2020; Kelcey et al., 2021; Zitzmann & Helm, 2021). Of note, when more than 10 waves of data are available, other multi-level models that make use of data in long format are likely more suitable than RI-CLPMs. For instance, dynamic structural equation modelling (DSEM) allows for the estimation of within-person autoregressive and cross-lagged effects (Hamaker et al., 2018). However, as of now, DSEM has only been used for *Between × Within* interactions. Second, to aid convergence time, measurement error variances were fixed to 0.2 for repeatedly measured variables rather than to 0. This means that observed variables were not completely separated into within- and between-person components with a small proportion of variance not being accounted for. Further research into methods that may aid model convergence as well as into model comparisons within latent variable interaction models is needed. As of yet, the decision on whether the inclusion of an interaction effect is warranted is based on evaluating their significance level (Asparouhov & Muthén, 2020); however, this does not offer clear insights into whether the inclusion of such effects leads to a meaningful improvement in model fit. Finally, while the MCS provides stratification variables and sampling and attrition weights, it was not possible to incorporate those as Mplus cannot yet account for complex sampling designs when using a Bayesian estimator.

## Conclusion

The incorporation of interaction effects into RI-CLPMs has strong potential for illuminating mechanisms of interest to developmental researcher as they decompose observed variables into within- and between-person components, thus, allowing for a much clearer interpretation of results. Such moderation analyses can be easily implemented using the SEM software Mplus. Together, these tools allow us to better isolate, estimate and interpret the complexities of within-person and between-person dynamics as they unfold over time.

## Supplementary Material

Supplementary Materials

## Data Availability

The University of London Centre for Longitudinal Studies owns the copyright for the Millennium Cohort Study (MCS) data used in this study. The MCS data are held/curated by the UK Data Service. Anyone wishing to use the MCS data (found at: https://discover.ukdataservice.ac.uk/series/?sn=2000031) must register and submit a data request to the UK Data Service at http://ukdataservice.ac.uk/. Additional terms and conditions are outlined here: https://www.ukdataservice.ac.uk/get-data/how-to-access/conditions.

## Availability of Code

Code is available on the Open Science Framework: https://osf.io/tjxrd/?view_only=3049a194340340f892dfe0ed055e040a.
