# Supplementary material for: Testing for Within × Within and Between × Within Moderation using Random Intercept Cross-Lagged Panel Models
Source: Struct Equ Modeling. Author manuscript; Available in PMC 2023 Nov 7. (PMC7615284; doi:10.31234/osf.io/wktrb)
Supplement: Supplementary Materials [file EMS190339-supplement-Supplementary_Materials.pdf]

Supplementary Materials

Table S1. Descriptive Statistics

**Variable**	***N***	***Mean***	***Sd***	***Min***	***Max***	***Skew***	***Kurtosis***
Conduct Problems Age 3	12217	2.798	2.054	0.000	10	0.735	0.268
Conduct Problems Age 5	13023	1.503	1.504	0.000	10	1.175	1.577
Conduct Problems Age 7	13655	1.398	1.553	0.000	10	1.387	2.300
Emotional Problems Age 3	12194	1.363	1.490	0.000	10	1.529	3.056
Emotional Problems Age 5	13005	1.386	1.597	0.000	10	1.514	2.638
Emotional Problems Age 7	13626	1.540	1.776	0.000	10	1.449	2.149
Peer Problems Age 3	12128	1.538	1.600	0.000	10	1.107	1.022
Peer Problems Age 5	12998	1.155	1.454	0.000	10	1.573	2.822
Peer Problems Age 7	13635	1.234	1.559	0.000	10	1.582	2.793
Cognitive Abilities	11516	57.943	30.377	0.200	100	-0.362	-1.153

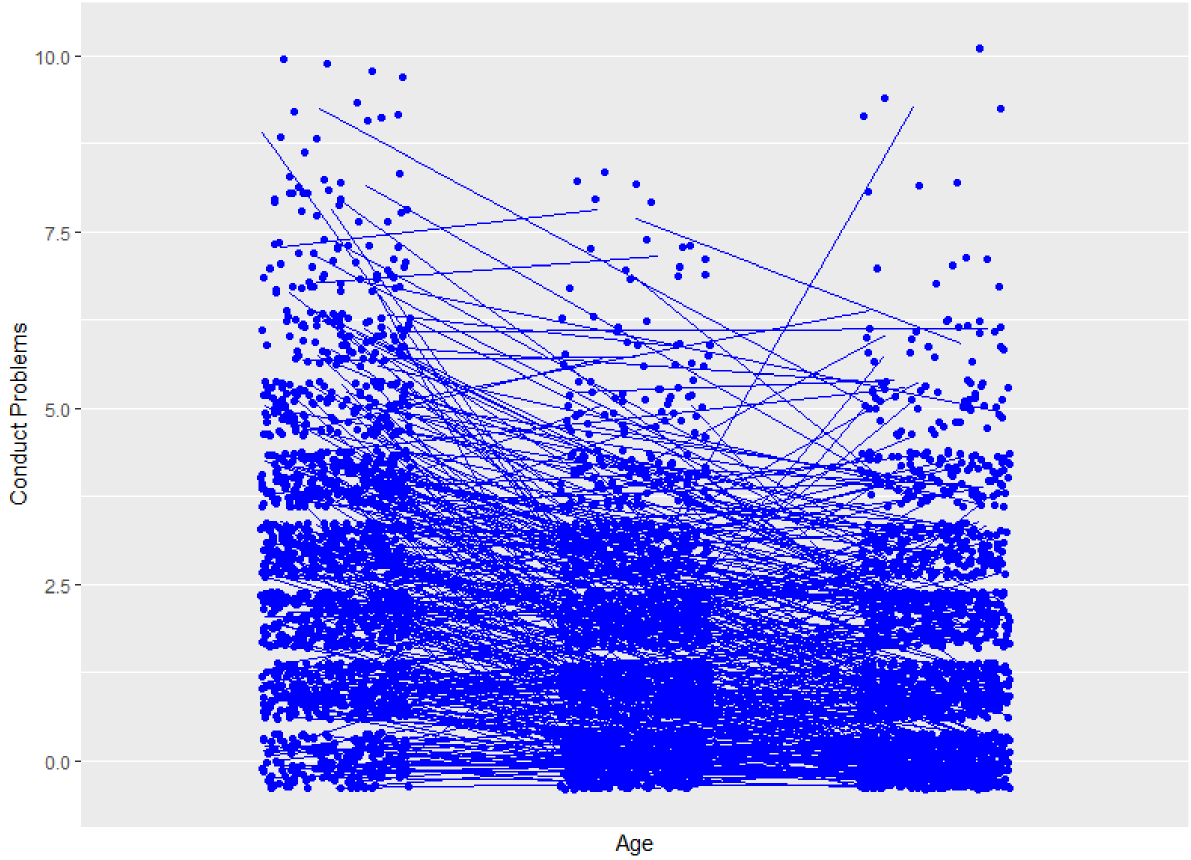

Figure S1. Trajectories of conduct problems in a random sub-sample of 5000 children.

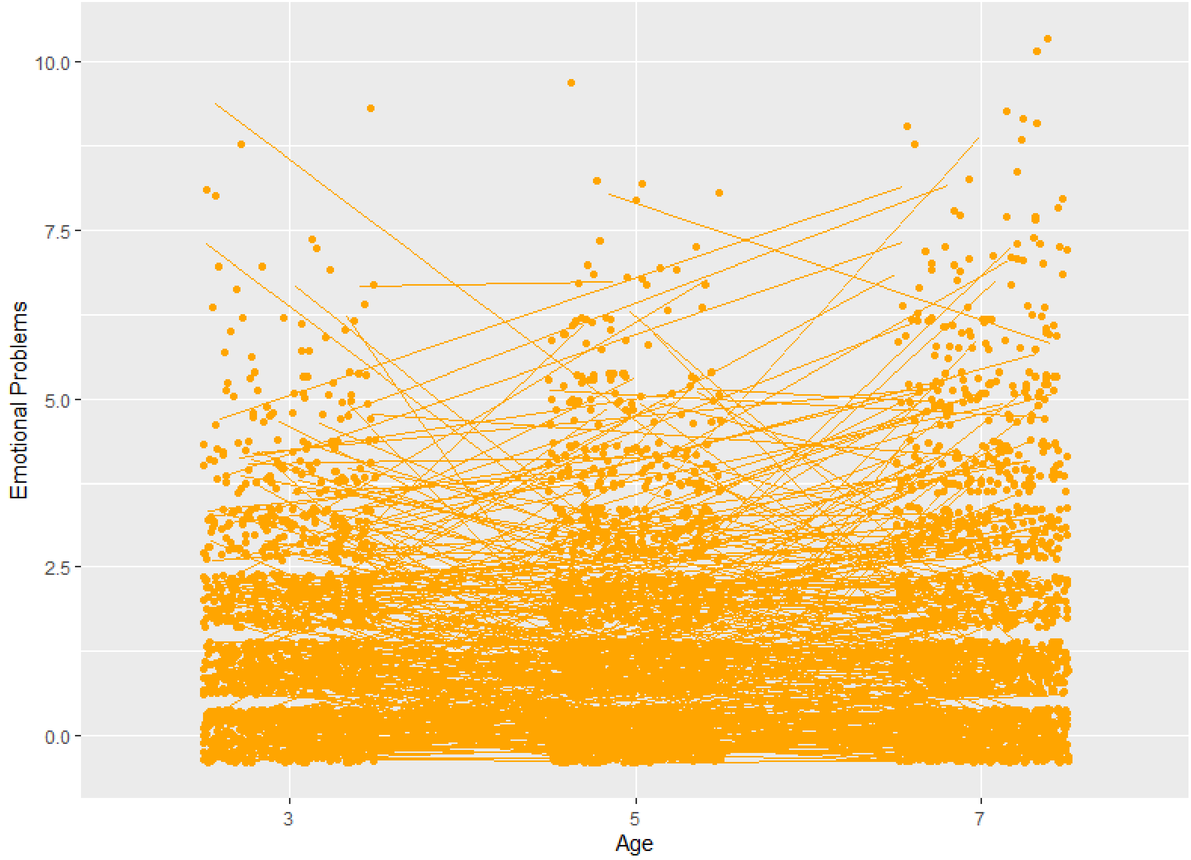

Figure S2. Trajectories of emotional problems in a random sub-sample of 5000 children.

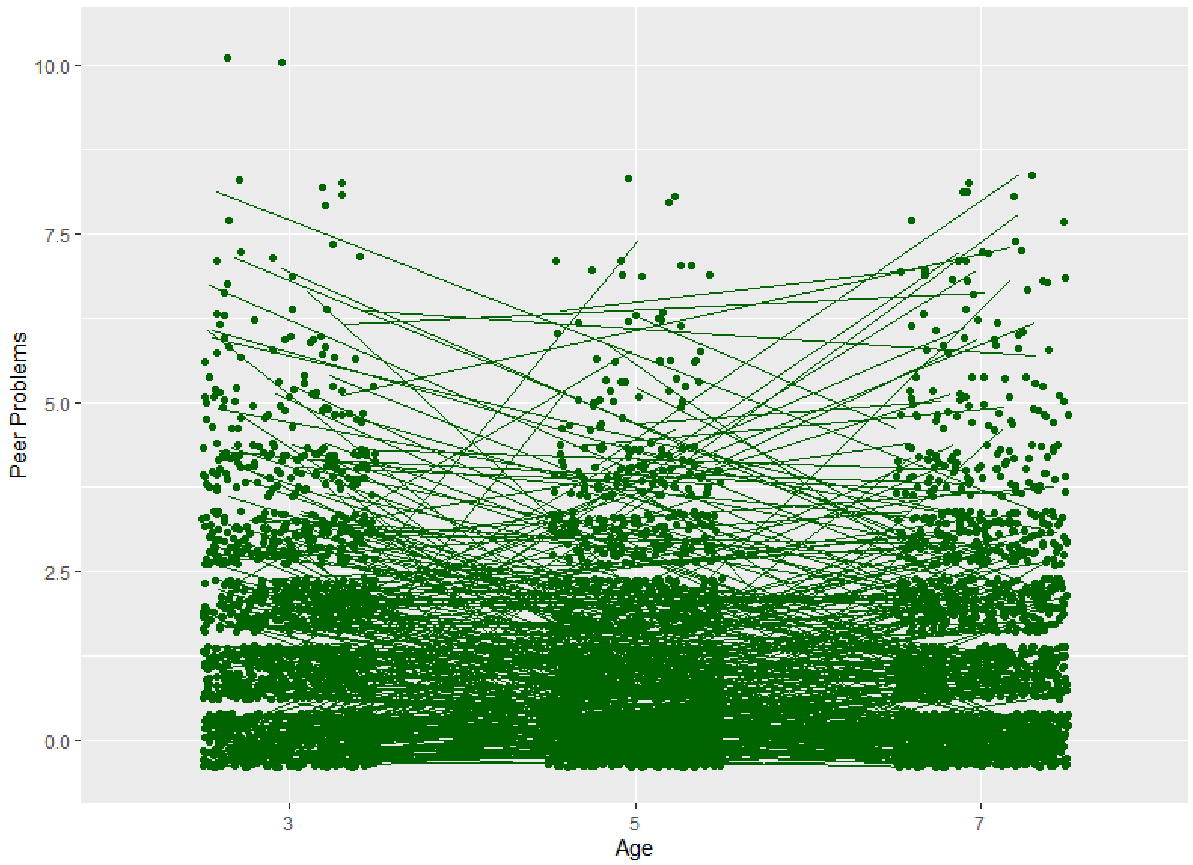

Figure S3. Trajectories of peer problems in a random sub-sample of 5000 children.

Table S2. Summary of Model Estimates.

**Variables**	**Baseline Model**	**Time-invariant Between x Within Model**	**Baseline + Peer Problems Model**	**Time-varying Between x Within Model**	**Within x Within Model**
**Within-Effects**	Estimate (CI_l/CI_u)	Estimate (CI_l/CI_u)	Estimate (CI_l/CI_u)	Estimate (CI_l/CI_u)	Estimate (CI_l/CI_u)
AR $EP_3 \rightarrow EP_5$	0.145* (0.08/0.21)	0.120* (0.05/0.19)	0.139* (0.05/0.20)	0.161* (0.11/0.22)	0.142* (0.08/0.20)
AR $EP_5 \rightarrow EP_7$	0.390* (0.35/0.43)	0.375* (0.34/0.41)	0.367* (0.33/0.40)	0.346* (0.31/0.38)	0.361* (0.32/0.40)
AR $CP_3 \rightarrow CP_5$	0.100* (0.08/0.13)	0.098* (0.07/0.12)	0.106* (0.08/0.13)	0.073* (0.05/0.10)	0.105* (0.08/0.13)
AR $CP_5 \rightarrow CP_7$	0.079 (-0.03/0.17)	0.074 (-0.03/0.18)	0.092 (-0.00/0.18)	-0.014 (-0.10/0.11)	0.086 -(0.01/0.18)
AR $PP_3 \rightarrow PP_5$	-	-	0.105* (0.06/0.15)	-	0.100* (0.60/0.14)
AR $PP_5 \rightarrow PP_7$	-	-	0.353* (0.31/0.40)	-	0.348* (0.30/0.39)
CL $EP_3 \rightarrow CP_5$	-0.007 (-0.05/0.03)	-0.018 (-0.06/0.02)	-0.015 (-0.06/0.03)	0.005 (-0.03/0.04)	-0.008 (-0.05/0.03)
CL $EP_5 \rightarrow CP_7$	0.093* (0.06/0.13)	0.095* (0.06/0.13)	0.068* (0.03/0.11)	0.088* (0.05/0.13)	0.074* (0.04/0.11)
CL $CP_3 \rightarrow EP_5$	0.097* (0.07/0.12)	0.067* (0.03/0.11)	0.085* (0.06/0.15)	0.076* (0.05/0.10)	0.086* (-0.01/0.18)
CL $CP_5 \rightarrow EP_7$	0.155* (0.35/0.43)	0.360* (0.24/0.48)	0.128* (0.07/0.18)	0.164* (0.08/0.24)	0.147* (0.08/0.21)
CL $EP_3 \rightarrow PP_5$	-	-	0.007 (-0.04/0.06)	-	0.018 (-0.03/0.07)
CL $EP_5 \rightarrow PP_7$	-	-	0.024 (-0.01/0.05)	-	0.030 (-0.00/0.06)
CL $CP_3 \rightarrow PP_5$	-	-	0.054* (0.03/0.08)	-	0.050* (0.02/0.11)
CL $CP_5 \rightarrow PP_7$	-	-	0.131* (0.08/0.19)	-	0.123* (0.03/0.07)
CL $PP_3 \rightarrow CP_5$	-	-	0.009 -(0.02/0.04)	-	0.006 (-0.02/0.04)
CL $PP_5 \rightarrow CP_7$	-	-	0.067* (0.02/0.11)	-	0.056* (0.01/0.10)
CL $PP_3 \rightarrow EP_5$	-	-	0.065* (0.03/0.10)	-	0.071* (0.04/0.11)
CL $PP_5 \rightarrow EP_7$	-	-	0.098* (0.06/0.14)	-	0.097* (0.06/0.14)

**Between*Within**	Estimate	Estimate	Estimate	Estimate	Estimate
Cog: CL $CP_3 \rightarrow EP_5$	-	0.001 (0.000/0.001)	-	-	-
Cog: CL $CP_5 \rightarrow EP_7$	-	-0.003* (-0.004/-0.001)	-	-	-
PP^B: CL $CP_3 \rightarrow EP_5$	-	-	-	0.037* (0.01/0.06)	-
PP^B: CL $CP_5 \rightarrow EP_7$	-	-	-	0.165* (0.10/0.24)	-
**Within*Within**	Estimate	Estimate	Estimate	Estimate	Estimate
$PP^W{}_3$: CL $CP_3 \rightarrow EP_5$	-	-	-	-	-0.010 (-0.03/0.09)
$PP^W{}_5$: CL $CP_5 \rightarrow EP_7$	-	-	-	-	0.105* (0.05/0.17)

Note. All estimates are unstandardised. CI_l = 2.5% Credible Interval, CI_u = 97.5% Credible Interval, EP = Emotional Problems, CP = Conduct Problems, PP = Peer Problems, ^{W} = Within Component, ^{B} = Between Component, CL = Cross-lagged effect, AR = Autoregressive effect, Numbers in subscript indicate age, * = significant based on credible intervals not containing zero. Due to long convergence times and computational limitations, the number of iterations was not double for the Time-varying Between x Within Model.
